# Advances in gait research related to Alzheimer’s disease

**DOI:** 10.3389/fneur.2025.1548283

**Published:** 2025-06-03

**Authors:** Shuding Yan, Xiaoping Yun, Qiang Liu, Zhenmei Hong, Yufan Chen, Shuijing Zhang

**Affiliations:** ^1^Department of Neurology, The Third School of Clinical Medicine (School of Rehabilitation Medicine) of Zhejiang Chinese Medical University, Hangzhou, Zhejiang, China; ^2^Department of Neurology, China Rehabilitation Research Center (CRRC), Beijing, China; ^3^Department of Neurological Rehabilitation, The Affiliated Rehabilitation Hospital of Zhejiang Chinese Medical University (Zhejiang Rehabilitation Medical Center), Hangzhou, Zhejiang, China

**Keywords:** Alzheimer’s disease, gait, cognitive deficit, gait analysis, machine learning

## Abstract

**Introduction:**

Alzheimer’s disease (AD) represents a degenerative condition affecting the nervous system, characterized by the absence of a definitive cause and a lack of a precise therapeutic intervention. Extensive research efforts are being conducted worldwide to enhance early detection methods for AD and to develop medications capable of effectively halting the initiation and progression of the disease during its early stages. Some current detection methods for early diagnosis are expensive and require invasive procedures. More and more evidence shows that gait is related to cognition. A deeper investigation into the intricate interplay between gait and cognition is necessary to elucidate their reciprocal influences and the temporal sequence of these interactions. In the future, it is hoped that with the results of clinical manifestations, neuroimaging, and electrophysiology, simple and objective gait analysis results can be used as an alternative biomarker for cognitive decline to diagnose dementia early.

**Research objective:**

This research offers a comprehensive scoping review of the contemporary landscape of clinical gait evaluation. It delineates the pertinent concepts of gait analysis and machine learning in AD and elucidates the intricate interplay between gait patterns and cognitive status.

**Methods:**

A comprehensive literature search was conducted within PubMed for all articles published until march 18, 2024, using a set of keywords, including “machine learning and gait “and “gait and Alzheimer.” original articles that met the selection criteria were included.

**Results and significance:**

A strong correlation exists between autonomous gait and cognitive attributes, necessitating further investigation into the selective interplay between gait and mental factors. Conversely, the gait information of Alzheimer’s disease (AD) patients can be captured using a 3D gait analysis system. Numerous gait characteristics can be derived from this gait data, and the early identification of AD can be facilitated by applying a graph neural network-based machine learning approach.

## 1 Introduction

A degenerative illness of the central nervous system, AD manifests itself in heightened cognitive impairment and behavioral abnormalities (1). The clinical manifestations are memory impairment, aphasia, apraxia, agnosia, visuospatial disturbance, abstract thinking and computational disorders, personality and behavior changes, gait disturbance, and decreased ability to perform daily living (1). AD not only causes patients to lose their ability to work, socialize, and take care of themselves, thus impacting their quality of life, but it also places a significant burden on their families and society (2, 3). Despite extensive research, there is currently no proven cure for AD, and its etiology remains unclear (4). The pathogenesis of AD is primarily based on hypotheses involving amyloid-beta (Aβ) deposition (5), tau phosphorylation (6), and changes in neurotransmitters (4). However, current diagnostic methods, such as cerebrospinal fluid assays and PET-CT, are expensive, invasive, and often inaccessible to many patients (7, 8). These limitations highlight the critical need for early and accurate diagnostic tools that are non-invasive, cost-effective, and widely available (9, 10). Moreover, existing treatments for AD are largely palliative, focusing on symptom management rather than disease modification (3, 11). These treatments offer modest benefits and fail to address the underlying pathological processes (12). As a result, there is an urgent need for novel therapeutic approaches that can effectively halt or slow AD progression, particularly in its early stages (13, 14).

Emerging evidence suggests that impairments in cognitive processes, including attention, executive functioning, and operational memory, are linked to decreased gait speed and instability in walking patterns (15). This association underscores the potential of gait assessment as a non-invasive biomarker for cognitive dysfunction in older adults (16, 17). Gait analysis may provide clinicians with a valuable tool for early intervention, potentially slowing the progression of AD and improving patients’ quality of life (1). While the association between gait and cognition in AD is well-established, a comprehensive understanding of the intricate interplay between specific gait parameters and cognitive domains remains elusive. Furthermore, the potential of utilizing advanced machine learning techniques to leverage gait data for early AD diagnosis has not been fully explored. This review aims to address these gaps by providing a comprehensive overview of the current landscape of clinical gait evaluation in AD, elucidating the relationship between gait patterns and cognitive status, and exploring the potential of machine learning in this context.

### 1.1 The definition and elements of gait

Gait reflects the manner or pattern of walking and is a biomechanical expression of the function of the central nervous system (18). Normal gait depends on the coordination of the central, peripheral, and musculoskeletal systems (18). When the coordination and balance of the above systems are damaged, it can lead to different degrees of walking difficulties and abnormal gait (19). Walking in the real world requires attention to various environmental features and recovery from postural disturbances (19). This process involves the cooperation of the cerebral cortex, subcortical, spinal cord, and peripheral neuroskeletal systems (19). Gait deviations are often associated with the pathological features of specific nerve, muscle, or bone diseases. Gait has many characteristics (spatiotemporal, kinematic, kinetic parameters, etc.). Some studies have developed various gait models based on gait parameters. The gait characteristics are categorized into different gait domains in the different gait models, e.g.,16 gait characteristics of older adults are classified into five domains, including speed, variability, rhythm, asymmetry, and postural control (20).

### 1.2 The definition and elements of gait analysis

**Figure 1:**
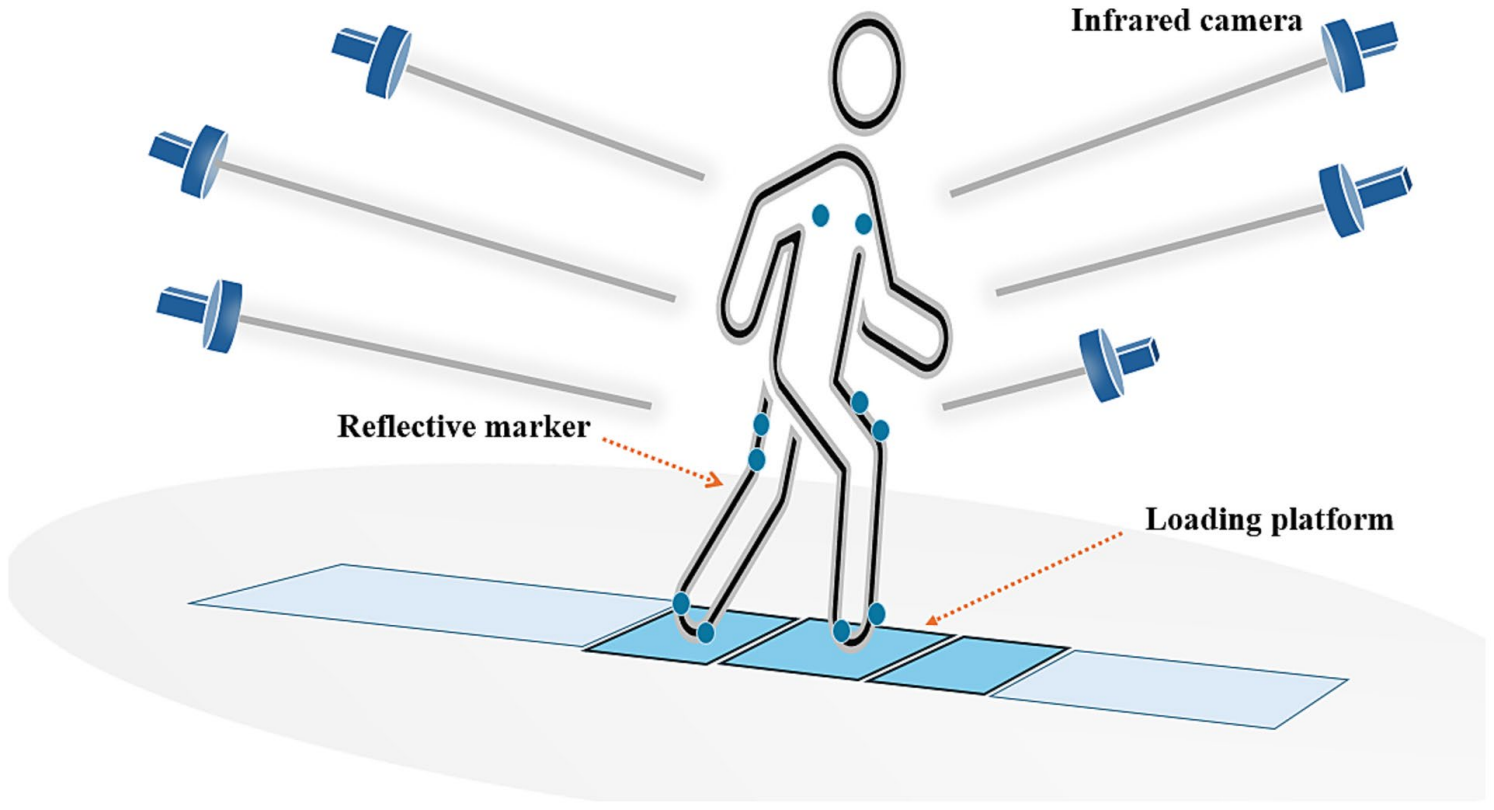
3D gait analysis, measurements made using a 3D photo tracking system (based on markers on the skin associated with bony landmarks) combined with a composite force measurement platform that quantifies joint kinematics (angles) and kinetics (muscles and other soft tissues exerting momentum on the joints).

Clinical gait analysis is the process of recording and interpreting biomechanical data during walking to identify abnormalities and guide clinical decision-making (21). It is commonly used for pre-treatment assessment, monitoring disease progression, and evaluating therapeutic outcomes (22). While human observation can detect deviations from normal gait, it may not identify the primary issues or compensatory strategies (23). Some studies have found that the pace measured with a stopwatch is lower than that measured using electronically-enabled devices such as wearable sensors (24). Therefore, the use of specialized gait analysis instruments can enhance the accuracy of assessments. At present, a consensus has been reached on the composition of clinical gait analysis (25), and the analysis methods mainly include simple wearable, running table, and three-dimensional analysis. The most advanced method of clinical gait analysis is 3D gait analysis (Figure 1) (26, 27). This technique uses a marker-based motion capture system with optoelectronic cameras to track reflective markers placed on anatomical landmarks. It combines data from force plates to quantify joint kinematics (angles) and kinetics (forces and moments) and often includes dynamic electromyography (EMG) and high-quality video recordings. The advantages of 3D gait analysis include high precision and the ability to provide detailed biomechanical insights (26, 27). However, it also has limitations, such as the need for specialized equipment, expertise in marker placement, and susceptibility to soft tissue artifacts and marker displacement. Additionally, the long preparation time and the requirement for a controlled environment can limit its accessibility for some patients. Gait analysis is most effective for analyzing repetitive gait patterns. For conditions like ataxia, where gait patterns are highly variable, or in cases where fatigue significantly affects gait, the utility of this method may be limited (28, 29).

### 1.3 The definition and elements of machine learning

Machine learning is an essential branch of artificial intelligence that employs a data-driven approach, where algorithms and models are trained to motivate computer systems to automatically extract potential patterns and regularities from large data sets, which in turn helps clinicians make decisions and predictions (30). Machine learning has recently emerged as a powerful tool in the field of medical imaging, including computer vision techniques that autonomously recognize and analyze image features. This methodology facilitates extracting advanced features and patterns from intricate neuroimaging datasets (31). The graph neural network (GNN), an influential machine learning paradigm, has recently garnered significant attention from researchers. In contrast to conventional neural networks, GNNs are better suited for managing unstructured data, encompassing social networks, molecular configurations, and the like. The core idea is to represent the data in the form of graphs and to carry out the information transfer and feature learning through the structure of the graphs and the properties of nodes (32, 33). Graph neural networks provide a new approach to gait research in AD patients by modeling gait data as a graph, with nodes representing key gait feature points and edges representing their relationships and interactions (34).

Gait prediction for AD is convenient and noninvasive and can be used in a new direction. Machine learning algorithms can process and analyze gait data to obtain more accurate and detailed gait assessment Results.

## 2 Methods

Drawing upon research examining the link between gait and cognition, this paper consolidates diverse clinical gait abnormalities and pertinent parameters associated with Alzheimer’s disease. The authors adopted a scoping review rather than a systematic review approach to address this broader research objective. A scoping review was chosen over a systematic review due to the wider scope of the research question and the need to map the extent of evidence on the topic. Scoping reviews are beneficial for identifying gaps in the literature and providing an overview of the existing research landscape. Unlike systematic reviews, which focus on specific questions and outcomes, scoping reviews allow for a more comprehensive and flexible approach.

### 2.1 Search criteria

A comprehensive literature search followed the Preferred Reporting Items for Systematic Reviews and Meta-Analyses (PRISMA) guidelines. The search was performed across multiple databases, including PubMed, Scopus, and Web of Science. The primary search terms used were “gait and cognition,” “gait and Alzheimer’s disease,” and “machine learning and gait.” Boolean operators (AND, OR) effectively combined these terms. No date limit was applied, and articles were updated until March 18, 2024. Following the initial screening, 29 pertinent publications were included, as detailed in Table 1.

**Table 1:** Summary of studies on gait and cognitive decline in Alzheimer’s disease.

References	Duration (years)	Research type	research purposes	Research results
Wilkins et al. (119)	1987	Case–control studies	Comparison of attention in patients with prefrontal lobe lesions and those with temporal lobe lesions	With low-frequency auditory and tactile stimuli, patients with prefrontal lesions showed significantly more execution errors than those with temporal lesions (*p* < 0.05)
Marquis et al. (120)	2002	Cohort studies	Independent predictors of dementia onset in normal older adults	In older persons at high risk of dementia, step speed is an independent predictor of dementia onset (*r* = 0.13).
Fellgiebel et al. (121)	2003	Case–control research	Hippocampal and white matter alterations in individuals with Mild cognitive impairment (MCI) and AD: a diffusion tensor investigation	MCI and AD patients have substantially greater left hippocampus diffusion tensor imaging (DTI) MD values than healthy controls (*p* = 0.002).
Montero-Odasso et al. (122)	2009	A randomized controlled study	Effect of donepezil on falls in patients with MCI	MCI patients in the donepezil group had faster gait speed after 1 month (*p* = 0.045), and MCI patients in the donepezil group had decreased gait variability after 4 months (*p* = 0.04), and the administration of donepezil significantly increased the gait speed of AD patients in both single- and dual-tasking, and decreased their gait variability single-tasking and multitasking (*p* < 0.05)
Buracchio et al. (123)	2010	Cohort studies	Gait modifications in MCI patients	Twelve years before MCI started, a slowing of stride was already noticeable. (*p* < 0.001)
Montero-Odasso et al. (124)	2010	Case–control studies	Changes in gait during dual tasking in patients with MCI	In the dual-task condition in both single-task and dual-task modes, the MCI group’s rate of stride time variability was noticeably higher than that of the standard control group (*p* = 0.002).
Yamada et al. (112)	2011	Cohort studies	The association between fall risk and dual-task expenses in healthy older individuals	In older persons who walk faster than average, dual-task cost predicts fall risk. (*p* < 0.001)
Taniguchi et al. (125)	2012	Cohort studies	An investigation on how Japanese elderly individuals’ gaits alter and their cognitive abilities	Gait frequency was not associated with cognitive level (*p* > 0.05)
Lord et al. (126)	2013	A descriptive study	Independent gait in the elderly	Classification of gait characteristics in old age into nine domains and 13 features
Verlinden et al. (127)	2013	Cohort studies	Gait patterns in normal aging	Gait variability is highly correlated with age and can represent the earliest gait abnormalities caused by aging
Callisaya et al. (128)	2012	Cohort studies	Association of magnetic resonance analysis of cranial structures with gait changes	Progressive white matter lesions and step speed slowing (*p* = 0.04) were linked with white matter atrophy (*p* = 0.001), step length reduction (*p* = 0.005), and rhythmic alterations (*p* = 0.001); hippocampus atrophy was associated with both outcomes (*p* = 0.006).
Koenraadt et al. (129)	2014	Cross-sectional studies	HBO in the prefrontal cortex during the gait cycle using the functional near-infrared spectroscopy (fNIRS) approach	Significant changes in HbO occur in the prefrontal cortex during all phases of normal gait.
Del Campo, et al. (130)	2016	Cross-sectional studies	Relationship in a dementia-prone elderly population between localized brain b-amylin (Ab) and gait speed	The anterior cingulate gyrus, occipital lobe, and nucleus accumbens all showed a link between Ab and reduced walking speed (*p* < 0.05).
Sakurai et al. (131)	2019	Cross-sectional studies	Cognitive Impairment in ApoE4 and Slow Gait Coexistence	The coexistence of ApoE4 and slow pacing reduced MMSE scores, and there was an interaction between the two [*F*(1.1074) = 18.4, *p* < 0.001]
Graff-Radford et al. (132)	2019	Prospective study	Relationship between cerebrospinal fluid biomarkers and cognition in Alzheimer’s disease	More were cognitively impaired after age and sex adjustment (27% vs. 9%; *p* = 0.005). Amyloid PET status was similar with and without high-convexity tight sulci (HCTS), but tau PET standard uptake value ratio (SUVR) was lower for those with HCTS after age and sex adjustment (*p* < 0.001).
Montero-Odasso et al. (44)	2020	Cohort studies	Dual memory and gait loss in older persons and the risk of dementia in the future	Dementia is most likely to occur in older people who experience concurrent decreases in gait speed and cognition (HR: 3.12, 95% Cl 1.23–7.93, *p* = 0.017).
Montero-Odasso et al. (133)	2020	Cross-sectional studies	Differences in step speed in dual-tasking older adults assessed using a stopwatch and electronic walkway	Step speeds tested using stopwatches were lower than those measured using electronically enabled devices (*p* < 0.001)
Tian et al. (79)	2020	Cohort studies	Neuroimaging features of elderly persons with memory loss and slowing gait speed	Elderly persons with impaired memory and gait speed essentially revealed lower volumes in the superior frontal gyrus, superior parietal lobule, thalamus, precuneus, and cerebellum (*p* < 0.01)
Chen et al. (134)	2020	Case–control studies	Machine learning classification models can predict different types of MCI patients.	The principal component analysis-support vector machine (PCA–SVM) model demonstrated better classification performance, with 91.67% accuracy and 0.9714 area under the receiver operating characteristic curve (ROC AUC), using the polynomial kernel function to classify PD–MCI and non-PD–MCI patients.
Zheng et al. (60)	2022	Case–control studies	Cognitive effects of dual-task gait analysis	In the MCI group, the cadence of both the walking motor task (WMT), the walking task (WT), and the walking calculation task (WCT) were significantly different. However, the cadence in the NE group only showed a significant difference between WMT and WT.
Seifallahi et al. (135)	2022	Case–control studies	Tools for assessing and diagnosing the development of AD	Using these features and a support vector machine classifier, the model classified the two groups with an average accuracy of 97.75% and an F-score of 97.67% for five-fold cross-validation and 98.68 and 98.67% for leave-one-subject out cross-validation. These results demonstrate the potential of our approach as a new quantitative complementary tool for detecting AD among older adults.
Huang et al. (136)	2022	A cross-sectional study	Memory deficits and increased risk of falls, potential neuroanatomical links to this association in older adults with amnestic Mild Cognitive Impairment (aMCI) and mild AD	Memory deficit was associated with increased fall risk in older people with aMCI and mild AD (*p* < 0.001). The atrophy of the medial temporal, frontal, and parietal lobes might mediate the association.
Wang et al. (137)	2022	Cohort studies	Assessment of brain function in patients with cognitive impairment based on fNIRS and gait analysis	There was no significant difference in only task between the cognitively impaired group and the cognitively healthy group; however, during the dual-task, compared with the results of task 1, there was a significant difference between the ROI area (t = 2.025, *p* = 0.048) and the gait of the dual-task (*p* < 0.05).
Collyer et al. (15)	2022	Cohort studies	Dual decline in cognition and gait speed with risk of dementia in older adults	Dual decline in gait speed and cognition was associated with an increased risk of dementia, with dual memory decliners showing the most significant risk (HR, 24.9; 95% CI, 16.5–37.6).
Bommarito et al. (138)	2022	A cross-sectional pilot study	The biological substrate of the Motoric Cognitive Risk (MCR) syndrome	MCR, especially in its motor component, is associated with lateral ventricular enlargement and microstructural damage of the sCR (*p* = 0.059) but not to amyloid (*p* = 0.550) or tau deposits (*p* = 0.582) or global white matter macroscopically detectable damage (*p* = 0.749).
Skillback et al. (73)	2022	A longitudinal study	Slowing gait speed preceded cognitive decline and correlated with brain amyloidosis.	Gait speed (*B* = 0.15, *p* = 0.024) decline precedes cognitive decline, is linked to Alzheimer’s pathology (*B* = 2.75, *p* = 0.067), and might be used for early detection of increased risk for dementia development.
Suzuki et al. (139)	2023	A clinical trial	A new balance capability index as a screening tool for mild cognitive impairment.	The new balance capability indicator, termed the visual dependency index of postural stability (VPS), was highly associated with cognitive impairment assessed by the Montreal Cognitive Assessment (MoCA). The area under the receiver operating characteristic (ROC) curve was more than 0.8, demonstrating high sensitivity and specificity (~80 and 60%, respectively).
Li et al. (45)	2023	Cohort studies	Temporal sequence between cognitive function and gait speed	There is a longitudinal bidirectional association between usual gait speed and both global cognitive function (*β* = 0.117, 95% CI 0.082–0.152; *p* < 0.001) and specific domains of mental intactness (*β* = 0.082, 95% CI 0.047–0.118; *p* < 0.001) and episodic memory (*β* = 0.102, 95%CI 0.067–0.137; *p* < 0.001) among Chinese older adults. Baseline global cognition is likely to have a stronger association with subsequent gait speed than the reverse pathway (χ12 = 6.50, P for difference = 0.01).
Lin et al. (23)	2024	A cross-sectional study	The integration of eye-tracking, gait, and corresponding dual-task analysis can distinguish cognitive impairment (CI) patients from controls.	A model based on dual-task gait, smooth pursuit, prosaccade, and anti-saccade achieved the best area under the receiver operating characteristics curve (AUC) of 0.987 for CI detection. In contrast, combined with phosphorylated tau 181 (p-tau181), the model discriminated mild cognitive impairment from controls with an AUC of 0.824.
Tuena et al. (140)	2024	Cohort studies	The prediction of future aMCI AD diagnosis by gait disorders and gait-related neuropsychological manifestations assessed by machine learning (ML)	The SVM algorithm achieved the best performance. The optimized training set performance attained an accuracy of 0.67 (sensitivity = 0.72; specificity = 0.60), improving to 0.70 on the test set (sensitivity = 0.79; specificity = 0.52). The ML model could quickly identify individuals at higher risk of AD.

### 2.2 Data screening and selection process

The screening process involved two independent reviewers who assessed the titles and abstracts of identified articles. Discrepancies were resolved through discussion, and inter-rater reliability was assessed using Cohen’s kappa coefficient. The kappa values were interpreted as follows: poor (<0.20), fair (0.20–0.40), moderate (0.40–0.60), good (0.60–0.80), and excellent (0.80–1.00). This process ensured the reliability and consistency of the selected studies.

## 3 Results

Traditionally, walking has been considered an autonomous behavior. However, this idea is now considered oversimplified (35). Gait is recognized as a marker of whole-brain health and an essential tool for predicting health status and survival in the elderly (36). A substantial body of research has shown that higher cognitive functions are necessary for safe and successful gait, replacing the notion that gait is merely a motor task (37). For example, studies have shown that executive function is closely related to gait variability (38). Specifically, decreased executive function is associated with increased stride time variability and reduced gait stability (39), which are significant predictors of falls and cognitive decline (40). Another study found that higher cerebral amyloid-*β* deposition is associated with increased double support time (41). These findings suggest that gait parameters can serve as early indicators of cognitive impairment. The conventional view is that gait and cognitive function decline parallel to aging, with both deteriorating over time to produce two common geriatric entities: falls and dementia (42). However, an emerging perspective suggests that cognitive decline predicts reduced mobility and fall risk, while reduced mobility and slow gait predict further cognitive deterioration (43). These phenomena are interrelated rather than merely concurrent. For example, a longitudinal study showed that older adults with early onset of reduced mobility, characterized by decreased walking speed, were more likely to experience clinically verifiable cognitive decline (15). Gait is a strong predictor of future cognitive impairment and dementia in older adults (15, 44, 45). Motor Cognitive Risk Syndrome (MCR) (46) refers to a syndrome that can predict dementia risk based on slow gait and subjective cognitive decline in older adults. Studies have shown that older adults with both gait speed decline and cognitive decline are at a higher risk of developing dementia (15, 45). Cross-sectionally, studies have identified partial associations between gait and cognition in normal aging and specific neurodegenerative diseases (e.g., Parkinson’s disease, Alzheimer’s disease), supported by neuroimaging studies (47, 48). For example, a comprehensive gait measurement study found that gait domains such as pace/turning and variability were strongly associated with attention and executive function (40). Another study showed that gait variability 39, particularly stride length variability, was strongly associated with executive and global cognitive function in community-dwelling older adults. These findings underscore the potential of gait analysis as a non-invasive biomarker for the early detection of cognitive decline.

Machine learning algorithms, particularly graph neural networks (GNNs), have shown significant potential in analyzing gait data for the diagnosis of Alzheimer’s disease (AD). These advanced techniques can process complex, non-Euclidean data and capture subtle patterns that traditional methods might miss (49). For example, GNNs have been used to analyze gait data by constructing graphs that represent the relationships between different gait parameters, such as stride length and step time variability (49). One notable application is the use of attention-based spatial–temporal graph convolutional networks (AST-GCN) (50), which can effectively capture the dynamic relationships between spatial and temporal features in gait data. This approach has been shown to improve the accuracy of AD diagnosis by identifying unique gait patterns associated with cognitive decline (51). Machine learning models, especially GNNs, can achieve higher diagnostic accuracy by leveraging complex patterns in gait data (52). These algorithms can automatically identify relevant features from raw gait data, reducing the need for manual feature engineering (53). Machine learning models can handle large datasets, making them suitable for analyzing extensive gait data from diverse populations (49). However, many machine learning models, including GNNs, operate as “black boxes,” making it difficult to interpret the reasoning behind their predictions (53). This lack of transparency can be problematic in clinical settings where interpretability is crucial (54). Effective training of these models requires large, high-quality datasets, which may not always be available (49). Additionally, the generalizability of these models to different environments or populations can be limited (55). Implementing and optimizing machine learning models, especially GNNs, require specialized technical expertise (54).

## 4 Discussion

### 4.1 Independent gait traits are linked to specific cognitive processes

Independent gait features are associated with discrete cognitive functions, and numerous studies have shown that the gait domain is inextricably related to the cognitive domain in AD. For example, in elderly patients with mild to moderate dementia, slowing gait speed is an early and specific change (56). The evidence for step speed as a predictor of whole-brain cognitive status is strong and effective in predicting declines in executive functioning (57) and processing speed; however, a direct predictive relationship between step speed and memory loss was not found. High gait variability may be a sensitive marker of prefrontal cortical control dysfunction during walking in patients with moderate AD and individuals with executive dysfunction (58). Gait asymmetry is one of the least studied variables, and current research suggests that it is not significantly associated with cognitive functioning (59). Several studies also failed to find any correlation between gait asymmetry and cognitive functioning (46). Several studies have identified an association between gait rhythm and memory decline (15). Still, there is no conclusive evidence on whether rhythm can be used as a predictor of whole-brain cognitive decline (60). Another study sees gait rhythm as a risk factor for dementia onset (16, 60). In patients with impaired cognitive function, the hippocampus may mediate the association between cognitive function and gait parameters and is strongly associated with cognitive decline (61).

Cognition is most closely related to gait speed among all the measured gait features. Due to its utility and reliable measurement characteristics (45), gait speed is commonly employed as an indicator of gait. The gait model encompasses 16 parameters, among which gait speed is the least specific yet most sensitive indicator. The deterioration in general gait is reflected, but the underlying reasons are not. Given that gait speed is the “final expression” of gait. Therefore, the association between gait speed and cognition may be more pronounced for this overall measure, providing a basis for understanding the broader relationship between gait and cognition and a platform for more specific inquiry. However, given the complexity of gait, which constitutes a multifaceted construct comprising numerous discrete attributes, a solitary outcome cannot adequately represent it. While gait speed exhibits pathological sensitivity, it cannot differentiate or mirror subtle and selective neuropathological alterations in gait (15). Conversely, gait variability offers an alternative measure of unstable strides and has garnered considerable attention in recent literature. Although gait speed and variability changes are not mutually exclusive, they provide different information. For example, gait variability (cross-step fluctuations in gait) is a more sensitive predictor of falls than gait speed in some neurodegenerative disorders and identifies AD (62).

In AD research, the link between gait and cognitive function has received much attention (63). The cognitive domain most closely associated with gait is executive function (64), primarily governed by centers in the frontal lobe, subcortical structures, thalamus, anterior cingulate gyrus, and basal ganglia. Pathological (65) and imaging studies (66) imply that the prefrontal cortex (PFC) is a central site of executive attention that triggers purposeful behaviors integral to daily life. During movement, the PFC drives executive attention processes that regulate gait (67, 68). Notably, gait speed in AD patients is closely related to the structure of specific brain regions (26). Studies have shown that gait speed is mainly associated with gray matter volume in the posterior temporal–parietal-occipital brain regions, including the lingual gyrus, fusiform gyrus, middle occipital lobe, post-central gyrus, precuneus, inferior temporal gyrus, and superior temporal gyrus (69). These regions are mainly responsible for essential functions such as visual perception, language, semantic memory processing, and multimodal sensory integration. Further studies found a significant correlation between slower gait speed in AD patients and smaller gray matter volume (GMV) in the medial temporal lobe and motor brain regions (70). This suggests that, in addition to executive function-related brain regions, structural changes in hindbrain regions also play an essential role in gait abnormalities in AD patients. While executive attention declines to some extent during normal aging (71), deficits in executive function are more pronounced in AD (58). The decline in PFC function leads to slower gait speeds and insufficient stride length (72). Therefore, a comprehensive understanding of the neural mechanisms underlying gait abnormalities in AD patients requires careful consideration of structural changes in executive function-related brain regions as well as hindbrain regions.

These findings provide important insights for clinical practice. First, gait analysis can be used as a potential screening tool for early detection of Alzheimer’s disease (AD) and its associated cognitive impairments (1). For example, parameters such as gait speed (73) and gait variability (1, 38) have high accuracy in distinguishing cognitively normal individuals from cognitively impaired patients, especially under dual-task conditions (74) [e.g., naming animal task (75)], where gait speed and gait variability (1) can more sensitively identify cognitively impaired patients. In addition, gait variability (73) has high specificity in identifying individuals with AD, making it potentially useful in clinical settings. Second, gait-based interventions may help improve cognitive function and slow disease progression. Studies have shown that multielement exercise (76) (e.g., aerobic training, muscle strength training, and gait training) can improve walking speed and stride length in patients with mild cognitive impairment (MCI). In addition, dual-task training, progressive strength, and functional training (75) can improve walking speed in patients with comorbid cognitive and motor impairments (76). Although large-sample clinical trials are needed to validate the interventional effects of these training on MCI progression, these preliminary findings suggest that gait interventions may become a promising nonpharmacological treatment that can help improve cognitive function and quality of life for patients.

### 4.2 Pathology and neural networks of gait-cognition associations

The association pattern between individual gait characteristics and distinct cognitive functions varies across different pathologies, and this specificity will enhance our understanding of their underlying common pathology and shared neural circuitry and facilitate the identification of temporal changes in these patterns. This specificity will also aid in identifying patterns of change at each time. Understanding potential correlations between gait and cognition is made possible by current knowledge of disease pathophysiology. For example, AD patients most commonly present with memory loss, mainly due to amyloid deposition in the entorhinal cortex and hippocampus (77), and a correlation has been found between hippocampal atrophy and gait changes, primarily reflected in slower speed and smaller step length (78). Some studies have revealed that elderly individuals experiencing declines in both gait speed and mental function predominantly demonstrate reductions in volume within the cerebellum, parietal lobe, thalamus, precuneal cortex, and superior frontal cortex (79); One study found a significant correlation between gait speed and Aβ (measured by amyloid PET) in the caudate nucleus, occipital cortex, precuneus, and anterior cingulate gyrus, hypothesizing that deposition of cerebral Aβ in these brain regions may be a potential mechanism by which slow gait speed (in the context of concomitant subjective cognitive deficits) is a strong predictor of future cognitive status (57, 80, 81). The exact mechanism is unclear, and both Aβ and tau may exert neurotoxic effects on the cognitive-motor network through different pathways at different time points in the disease (82). It has also been found that individuals carrying the ApoE4 genotype may have lower MMSE scores compared to slow walkers. There is an interaction between the ApoE4 genotype and slow walkers, which may affect cognitive functioning (83, 84).

That cognition and gait share common neural substrates are also supported by several studies, e.g., the neurotransmitter acetylcholine has been shown to play an essential role in cognitive function as well as gait control and balance (85), and acetylcholine (Ach) has been linked to attentional processes in the PFC (86), which is related to gait speed (87). Two additional studies (88, 89) that assessed the impact of cholinesterase inhibitors on gait performance revealed that donepezil and galantamine improved AD patients’ ability to adapt their gait patterns to tasks requiring attention and increased gait speed.

The coordination of neuronal networks connecting to the prefrontal cortex may be necessary for gait, and there is a reciprocal relationship between these two structures (90, 91). Gait is involved in two distinct but interacting neural pathways: the motor route and the cognitive pathway (92, 93). Both cognitive and motor pathways are controlled by brain areas such as frontal lobes, cerebellum, and basal ganglia that collectively interact to exert governance and control over executive function and intentionality of movements that require anticipation and the prediction of movement of others (94). For example, prefrontal cognitive and motor pathways require bidirectional communication to execute the movement, and gait impairment will occur if dysfunction exists in either structure. The prefrontal cognitive pathway and motor pathway in Lewy body dementia (LBD) and AD, respectively, are affected at different times, which leads to different cognitive performance, as well as gait impairment. Due to the breakdown of motor networks (such as the basal ganglia and associated networks), significant gait impairments appear early in LBD (95, 96). It has been found that in Lewy body dementia (LBD) and AD, prefrontal cognitive and motor pathways are affected at different stages, leading to different cognitive performance and gait impairments (97–99). In LBD, significant gait impairment occurs early due to dysfunction of motor networks (e.g., basal ganglia and related networks) (100, 101).

In contrast, cognitive networks have relatively better control over gait, allowing for a more significant shift from motor function to cognitive tasks (101). In contrast, in AD, where early pathologic changes are predominantly found in the cortex and do not affect the basal ganglia until late in the disease, cognitive control of gait may diminish earlier, leading to an increased reliance on motor networks to facilitate and regulate gait (100). Thus, gait deficits are more associated with motor deficits in AD, whereas the relationship between gait deficits and prefrontal-mediated cognitive functions (e.g., executive functions) is more pronounced in LBD (99, 102).

Future studies should explore the association of neural mechanisms between gait and cognitive function, including the effects of Aβ and tau proteins (103) on the cognitive-motor network and the dynamics of neurotransmitters (104), such as acetylcholine, in disease progression. Meanwhile, studies combining multimodal biomarkers (103) (e.g., Aβ-PET, tau-PET, cerebrospinal fluid markers, and genetic markers) will contribute to a more comprehensive understanding of the link between the two and provide new avenues for early identification and monitoring of Alzheimer’s disease. In addition, the development of graph neural network (GNN)-based multimodal data analysis models (105) can better reveal the neural network characteristics of gait and cognitive functions and their dynamics in the disease. Future large-scale clinical trials are needed to validate the long-term effects of gait interventions (e.g., multi-element exercise and dual-task training) on cognitive function and disease progression and to explore their applicability at different disease stages. Ultimately, the development of early screening tools based on gait characteristics and personalized treatment strategies combining biomarkers and neuroimaging will provide new ideas and approaches to slow the progression of Alzheimer’s disease.

### 4.3 Early evaluation of the connection between cognition and movement in a dual-task paradigm

**Figure 2:**
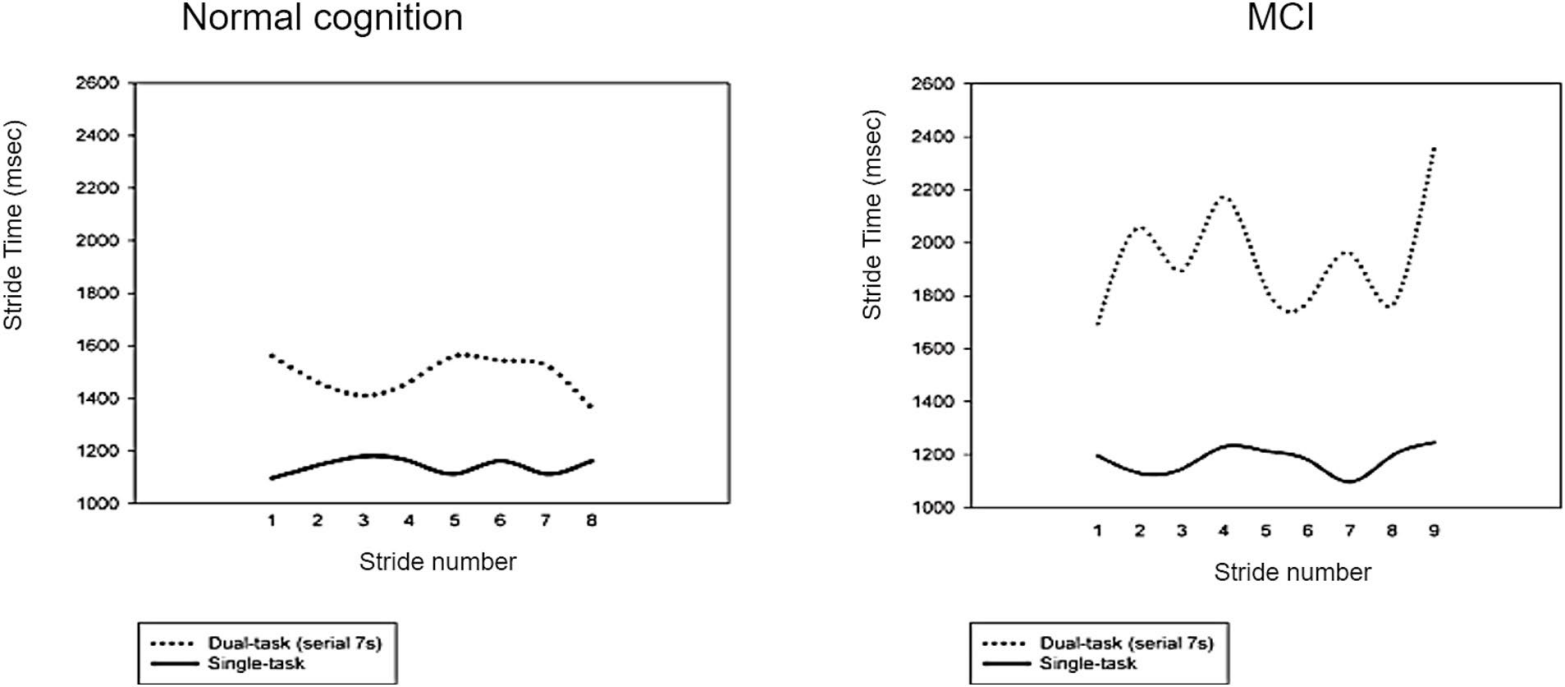
Comparison of changes in gait variability between cognitively ordinary people and MCI patients in single-task and dual-task modes reveals (112).

The ability of the cognitive system to manage movement is mirrored in gait under single-task settings. Thus, it is anticipated that changes in cognitive functioning will be reflected in changes in gait performance. Cognitive regulation of gait is reduced in MCI and early stages of dementia patients, but because the motor system compensates for cognitive deficits due to aging and pathology, changes in gait are not evident in single-task test conditions. This led to the groundbreaking study of gait in the dual-task “talk and walk” mode (106). We can generally only retain limited information in working memory (WM). In dual-task situations, individuals need to walk while performing another task that requires attention. Performing two tasks concurrently entails brain activity of greater complexity, necessitating coordination among cortical areas to manage the interconnected regions needed for executing each component task (107). Consequently, both walking and cognitive performance may undergo alterations, and they are frequently juxtaposed against cognitive or walking benchmarks in single-task scenarios to evaluate the modifications that occur (108). A fundamental premise of dual-tasking posits that two tasks executed simultaneously interfere with one another and vie for cortical resources (109). For instance, a decrement in gait speed may be construed as an augmentation in the cortical attentional demand during ambulation (107). The dual-task paradigm offers a practical and sensitive way to evaluate fall risk and motor-cognitive interactions without being neither pricy nor invasive. Dual-task costs discovered in gait evaluations may indicate mild brain injury (110) and are associated with attentional and executive function efficiency (111). One study comparing changes in gait variability between cognitively normal individuals and MCI patients in single-task and dual-task modes found no difference between the two in the single-task mode. Still, MCI patients in the dual-task mode showed a significant increase in gait variability (112) (Figure 2). Based on these findings, patients with mild cognitive impairment (MCI) and those in the initial stages of dementia exhibit diminished cognitive regulation of their gait performance. These disturbances are not noticeable under the single-task test conditions, but differences may be noticed under the dual-task condition. A study involving 1,038 elderly adults emphasized the sensitivity and predictive power of dual-task assessments in individuals with a generally normal gait. The results indicated that dual-tasking was a more frequent predictor of falls than single-tasking among those walking at speeds of 95 cm/s or above (112).

Recent advancements in gait analysis and machine learning offer promising avenues for the early detection and management of Alzheimer’s disease (AD). These findings have significant potential clinical implications, particularly in using gait analysis as a screening tool and developing gait-based interventions to improve cognitive function and slow disease progression.

## 5 Limitations

To date, selective associations between independent gait and cognitive traits have not been comprehensively examined, and most studies have focused on temporal–spatial parameters of gait, with fewer studies on kinematics, kinetics, surface EMG, etc. Additionally, despite the prevalent use of the dual-task paradigm in research to explore the associations between gait and cognition, discrepancies in findings have been documented, which can be attributed to methodological issues(e.g., various concurrent activities, accounting for baseline task demands and multiple approaches to computerized dual-task interference analysis) (113, 114). Along with these contradictions, the dual-task approach’s underlying cognitive nature remains unknown. It does not consider how baseline cognition affects gait, making pinpointing specific underlying brain correlates challenging. It is important to note that both voice analysis (115) and curve walking (116) are expected to be an affordable, easy-to-use, and highly accurate method of detecting early-onset dementia and to play an active role in the early identification of mild cognitive impairment (MCI), which opens up new ideas and potential complementary avenues of research in the field of gait and cognition. Suppose there is a selective link between gait and cognitive factors. In that case, the selective relationship between their intermediate neuropathology and neural networks must be investigated further. Still, current evaluations in pathology cohorts are particularly limited. Sample sizes tend to be minor, albeit with some exceptions (28), and more longitudinal studies that can draw causal conclusions are needed to elucidate the neural mechanisms underlying this association. As only a few research have shown that cognition is a predictor of changes in the gait domain, the issue of reverse causation must also be considered (15, 117). These findings show that locomotion and cognition have a complicated relationship, and they support the need for a thorough examination of both to understand how they interact and how their processes unfold through time.

## 6 Conclusion

**Figure 3:**
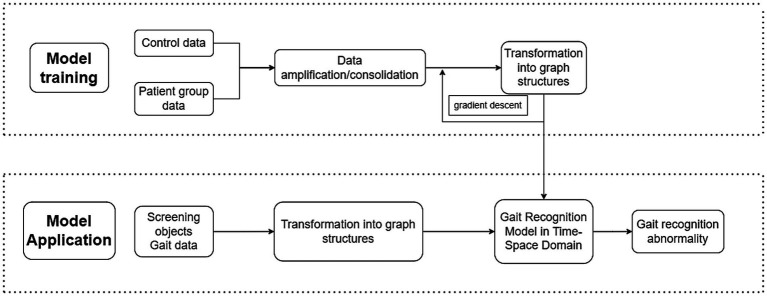
The training and application path of the neural network model.

In the future, based on the results of simple and objective gait analysis, it may be possible to incorporate kinematic and kinetic parameters and surface EMG parameters to investigate further and utilize dual-task methods to assess gait, to enhance gait’s ability to predict cognitive decline and to combine gait parameters with magnetic resonance (MRI), PET-CT, near-infrared techniques (fNIRS), electrophysiology (EEG, EMG), and other techniques. Analytical comparisons were performed using computer models to validate the role of gait as a surrogate biomarker of cognitive decline (118). Focusing on body coordination, training graph neural networks to learn the pattern differences between different gaits and realizing an accurate comparison between the gait of AD patients and ordinary people (Figure 3) can not only provide a deeper understanding of gait mechanisms but also is expected to provide new tool indicators for disease diagnosis and monitoring.
